# Delta‐He as a Novel Predictive and Prognostic Biomarker in Patients With NSCLC Treated With PD–1/PD‐L1 Inhibitors

**DOI:** 10.1002/cam4.70826

**Published:** 2025-04-05

**Authors:** Green Hong, Song‐I Lee, Da Hyun Kang, Chaeuk Chung, Hee Sun Park, Jeong Eun Lee

**Affiliations:** ^1^ Division of Allergy, Pulmonary, and Critical Care Medicine, Department of Internal Medicine Chungnam National University Hospital, Chungnam National University School of Medicine Daejeon Republic of Korea

**Keywords:** Delta‐he, immune checkpoint inhibitors, NSCLC, PD‐1/PD‐L1 inhibitors

## Abstract

**Background:**

Lung cancer treatment has rapidly advanced, particularly with immune checkpoint inhibitors (ICIs) targeting PD‐1 and PD‐L1. However, there are variable responses, such as immune‐related adverse events. Several factors predicting the prognosis of lung cancer ICI treatment have been studied so far, but they have limitations, leaving an unmet need. This study aims to investigate delta‐He, a novel marker reflecting the iron availability and inflammation through the difference in hemoglobin content between reticulocytes and erythrocytes, as a potential prognostic factor in patients with NSCLC undergoing PD‐1/PD‐L1 inhibitor therapy.

**Methods:**

This research was conducted at Chungnam National University Hospital, analyzing 79 advanced NSCLC patients treated with PD‐1/PD‐L1 inhibitors. The study population had a mean age of 70 years, with the majority being male (82.3%) and former or current smokers (84.8%). Blood samples collected before therapy initiation were examined for hematological parameters, including delta‐He, using Sysmex XN‐550 and XN‐20 analyzers. The study employed receiver operating characteristic (ROC) curves and Kaplan–Meier curves for the statistical analysis, using SPSS version 26 (IBM Corp., USA) and MedCalc version 22 (MedCalc Software Ltd., Belgium) to evaluate the sensitivity and specificity of delta‐He, and to analyze progression‐free survival (PFS) and overall survival (OS).

**Results:**

The study revealed that delta‐He is a significant prognostic marker in patients with NSCLC treated with PD‐1/PD‐L1 inhibitors. A delta‐He cutoff value of 3.3 pg was identified based on ROC analysis. Patients with high delta‐He values (> 3.3 pg) showed significantly longer median PFS (9.6 vs. 3.0 months, *p* = 0.024) and OS (not reached vs. 12.2 months, *p* = 0.010) than those with low values. The high delta‐He group also had higher objective response rate (41.4% vs. 26.0%) and disease control rate (86.2% vs. 52.0%). Multivariate analysis highlighted higher delta‐He (> 3.3 pg), along with other factors such as FEV1 and smoking status, as important predictors of survival, underscoring its potential role in guiding therapeutic decisions of ICIs in NSCLC (PFS: HR 0.874, 95% CI 0.264‐0.874, *p* = 0.016; OS: HR 0.327, 95% CI 0.150‐0.715, *p* = 0.005).

**Conclusion:**

Our study shows delta‐He as a promising prognostic biomarker for lung cancer patients treated with PD‐1/PD‐L1 inhibitors, highlighting its potential to guide therapeutic decisions and improve patient management in a non‐invasive manner. Further research is necessary to validate delta‐He's predictive and prognostic value across broader populations and in combination with other biomarkers, emphasizing its role in advancing personalized oncology.

## 1 Introduction

Lung cancer represents a paradigm of oncological evolution, experiencing swift and pivotal shifts in therapeutic strategies [1]. Since their introduction into clinical oncology, immune checkpoint inhibitors (ICIs), specifically programmed cell death 1 (PD‐1) and programmed cell death ligand 1 (PD‐L1) inhibitors, have led to significant breakthroughs in the management of lung cancer and are notably enhancing patient survival [2]. However, immunotherapy shows highly variable responses among patients. Although some patients achieve complete remission, others show paradoxical responses such as pseudoprogression or hyperprogression [3]. Additionally, immune‐related adverse events (irAEs) are significant factors closely associated with patient prognosis and require attention [4]. The need for reliable indicators to optimize therapeutic choices and patient outcomes is emphasized by the diverse immunotherapeutic landscape.

Several biomarkers predict the efficacy of immunotherapy in lung cancer. PD‐L1 expression in tumors is predictive of a higher response rate to ICI therapy in patients with non‐small cell lung cancer (NSCLC) [5, 6]. However, standardization and interpretation are difficult because of tumor heterogeneity, which limits its completeness [7]. Tumor mutation burden (TMB) [8] and tumor‐infiltrating lymphocytes (TILs) [9] have also been evaluated as biomarkers, but they require tissue testing; however, tissue testing cannot be performed if enough tissue is not available. To overcome these limitations, soluble PD‐L1 levels [10], blood‐based TMB values [11], and cytokines such as IL‐6 [12] have been investigated as alternative biomarkers that can be tested in the blood. However, there is still an unmet need.

Delta‐He is defined as the calculated difference between the mean hemoglobin (Hb) value of reticulocytes (RET‐He) and the mean Hb value of erythrocytes (RBC‐He). Reticulocytes have a higher Hb content compared to mature erythrocytes, resulting in the delta‐He values generally being positive [13]. RET‐He serves as an indicator of the extent of iron incorporation into erythroid progenitor cells over 2–4 days, indicating the level of functionally available iron. Delta‐He responds rapidly to changes in iron levels and reflects the availability of iron for erythropoiesis within a short span of 1–2 days [14, 15].

Delta‐He, as mentioned earlier, is a potential marker for iron availability and has been proposed as an indicator of inflammation. This relationship is due to the close link between iron homeostasis and inflammation, which is primarily mediated by hepcidin. Hepcidin mediates changes in iron metabolism, leading to reduced iron absorption from the intestine and redirection of iron from the bloodstream to the reticuloendothelial system and liver [16, 17]. The effectiveness of delta‐He as a predictive marker has been investigated in various patient groups, including patients on peritoneal dialysis [18, 19] and those undergoing surgeries [20]. However, delta‐He has been inadequately explored in the context of cancer, whereas the iron metabolism‐related marker, RET‐He, has primarily been evaluated in the context of cancer‐related anemia [21]. This study aims to evaluate the potential of delta‐He as a significant predictive and prognostic factor in non‐small cell lung cancer (NSCLC) patients treated with PD‐1/PD‐L1 inhibitors.

## 2 Patients and Methods

This study included patients with advanced NSCLC treated with PD‐1/PD‐L1 inhibitors at Chungnam National University Hospital (CNUH) between June 2021 and April 2023. Patients were included if they were treated with intravenous nivolumab (240 mg every 2 weeks), pembrolizumab (200 mg every 3 weeks), or atezolizumab (1200 mg every 3 weeks). Patients were enrolled regardless of whether they received ICI monotherapy or combination therapy. Treatment was continued until patients experienced serious adverse events, and investigator‐assessed disease progression was confirmed. Patients expected to experience clinical benefits were allowed to continue treatment beyond radiological disease progression.

This study retrospectively reviewed medical records; however, patient samples were collected prospectively in accordance with the Declaration of Helsinki and Good Clinical Practice guidelines, and the study was approved by the Institutional Review Board of CNUH (2020–10‐077). Written informed consent was obtained from all patients prior to their participation in the study.

Blood samples from patients were selected only if they were collected before PD‐1/PD‐L1 inhibitor administration. Collected blood samples were immediately placed on ice or were refrigerated until testing in the laboratory.

All hematological parameters, including the delta‐He value, used in this assay were analyzed using Sysmex XN‐550 and Sysmex XN‐20 (Sysmex Inc., Kobe, Japan).

PD‐L1 expression levels were determined using a qualitative immunohistochemical staining method; particularly, the in vitro diagnostic PD‐L1 IHC SP263 assay (Ventana Medical Systems, Tucson, AZ, USA). To categorize PD‐L1 expression, three distinct groups were established based on thresholds of 1% and 50%: < 1% expression was considered negative; 1%–49% was categorized as low; and > 50% was categorized as high.

Using computed tomography, patients treated with pembrolizumab or atezolizumab and patients treated with nivolumab were assessed for response every three and four cycles, respectively. Responses to ICI treatment were assessed using the Response Evaluation Criteria in Solid Tumors version 1.1. Clinical benefits were defined as disease control rate (DCR), including partial response (PR), stable disease (SD), and complete remision (CR). Progression‐free survival (PFS) was defined as the time from the date of the first ICI treatment to the date of documented progression or death from any cause. Overall survival (OS) was measured from the date of the first ICI treatment to the date of death or the last day of follow‐up.

Conventional receiver operating characteristic (ROC) curves and area under the curve (AUC) were used to calculate the sensitivity and specificity of delta‐He analysis. The optimal cutoff value was determined as the point at which the Youden index was maximized by the ROC curve. Chi‐squared and independent *t*‐tests were used to analyze differences in the patients' clinicopathological data. Survival was estimated using the Kaplan–Meier method, and survival rates were compared using the log‐rank test. Statistical significance was set at *p* < 0.05. Univariate and multivariate Cox regression analyses were performed to investigate the associations between PFS, OS, and various factors. All covariates included in the multivariate analysis were selected based on their clinical relevance and statistically significant associations with PFS and OS in the univariate analyses. Statistical significance was defined as *p*‐value < 0.1 for the univariate analysis and < 0.05 for the multivariate analysis. SPSS version 26 (IBM Corp., Armonk, NY, USA) and MedCalc version 22 (MedCalc Software Ltd., Ostend, Belgium) were used for all the statistical analyses.

## 3 Results

### 3.1 Patient Baseline Characteristics

From June 2021 to April 2023, 103 patients were enrolled, of which 24 patients with blood samples obtained after immunotherapy initiation were excluded from the study. These patients were excluded to prevent the influence of immunotherapy on delta‐He measurements.

Table 1 provides an overview of the baseline demographic and clinical characteristics of the study participants. The study cohort comprised 79 patients with advanced NSCLC, with a mean age of 70.0 years (± 8.4). The majority of patients were men (82.3%), and a significant proportion of the patients had a history of smoking, with 84.8% classified as former or current smokers. Disease staging at treatment initiation revealed a predominant distribution in stages IVA (58.2%) and IVB (30.4%).

**TABLE 1:** Patient characteristics and Treatment outcomes.

Variables	Value
Age (years)	70.0 ± 8.4
Sex	
Male	65 (82.3)
Female	14 (17.7)
Smoking status	
Never	12 (15.2)
Former/Current	67 (84.8)
Staging at treatment	
IIIA	1 (1.3)
IIIB	5 (6.3)
IIIC	3 (3.8)
IVA	46 (58.2)
IVB	24 (30.4)
Comorbidities	
DM	23 (29.1)
HTN	37 (46.9)
COPD	21 (26.6)
Asthma	4 (5.1)
ILD	1 (1.3)
Histology	
Adenocarcinoma	43 (54.4)
Squamous carcinoma	33 (41.8)
Other	3 (3.8)
Driver mutation	
EGFR mutation	2
KRAS mutation	7
ALK rearrangement	1
ROS1 mutation	1
BRAF mutation	1
PD‐L1 (sp263)	
High (≥ 50%)	19 (24.1)
Low (1 ~ 50%)	14 (17.7)
No (≤ 1%)	43 (54.4)
Number of prior regimen	
0	35 (44.3)
1	38 (48.1)
2 or more	6 (7.6)
Treatment method	
Monotherapy	58 (73.4)
Combination therapy	21 (26.6)
Concomitant RT	19 (24.1)
Presence of irAE	19 (24.1)
Agent	
Nivolumab	4 (5.1)
Pembrolizumab	31 (39.2)
Atezolizumab	44 (55.7)
Initial response to treatment	
PR	22 (27.9)
SD	29 (36.7)
PD	27 (34.2)
Best response to treatment	
PR	25 (31.7)
SD	26 (32.9)
PD	27 (34.2)

*Note:* Data are presented as mean ± standard deviation or number (%), unless otherwise indicated. One patient died four days after initiating therapy and was therefore not evaluable for treatment response.

Abbreviations: ALK, anaplastic lymphoma kinase; BRAF, B‐Raf proto‐oncogene, serine/threonine kinase; COPD, chronic obstructive pulmonary disease; DM, diabetes mellitus; EGFR, epidermal growth factor receptor; HTN, hypertension; ILD, interstitial lung disease; irAE, immune‐related adverse events; KRAS, kirsten rat sarcoma viral oncogene homolog; PD, progressive disease; PD‐L1, programmed death‐ligand 1; PR, partial response; ROS1, c‐ros oncogene 1; RT, radiation therapy; SD, stable disease.

Common comorbidities included diabetes mellitus (DM) (29.1%), hypertension (46.9%), and chronic obstructive pulmonary disease (COPD) (26.6%). Adenocarcinoma (54.4%) and squamous carcinoma (41.8%) were the predominant histological subtypes. Molecular profiling identified *EGFR* mutations in 2 patients; *KRAS* mutations, 7; *ALK* rearrangement, 1; *ROS1* mutation, 1; and *BRAF* mutation, 1. PD‐L1 expression was variable, with 19 (24.1%) exhibiting high expression (≥ 50%); 14 (17.7%), low expression (1% – 50%), and 43 (54.4%), no expression (≤ 1%).

The distribution of prior treatment regimens was 44.3% with no prior regimens, 48.1%, 1; and 7.6% ≥ 2. Treatment modalities included monotherapy in 73.4% of cases and combination therapy in 26.6%. Concomitant radiation therapy was administered to 24.1% of patients. IrAEs occured in 24.1% of cases.

The three main agents administered were nivolumab (5.1%), pembrolizumab (39.2%), and atezolizumab (55.7%). Initial treatment responses demonstrated PR in 27.9%; SD, 36.7%; and, progressive disease (PD), 34.2%. The best treatment responses were PR in 31.7%; SD, 32.9%; and PD, 34.2%.

### 3.2 Predictive Value of Baseline Delta‐He

Delta‐He values ranged from −5.2 to 7.4 pg, with a mean of 2.4975 ± 2.00454 pg. These descriptive statistics provided a snapshot of the distribution of delta‐He values within the examined cohort.

**FIGURE 1:**
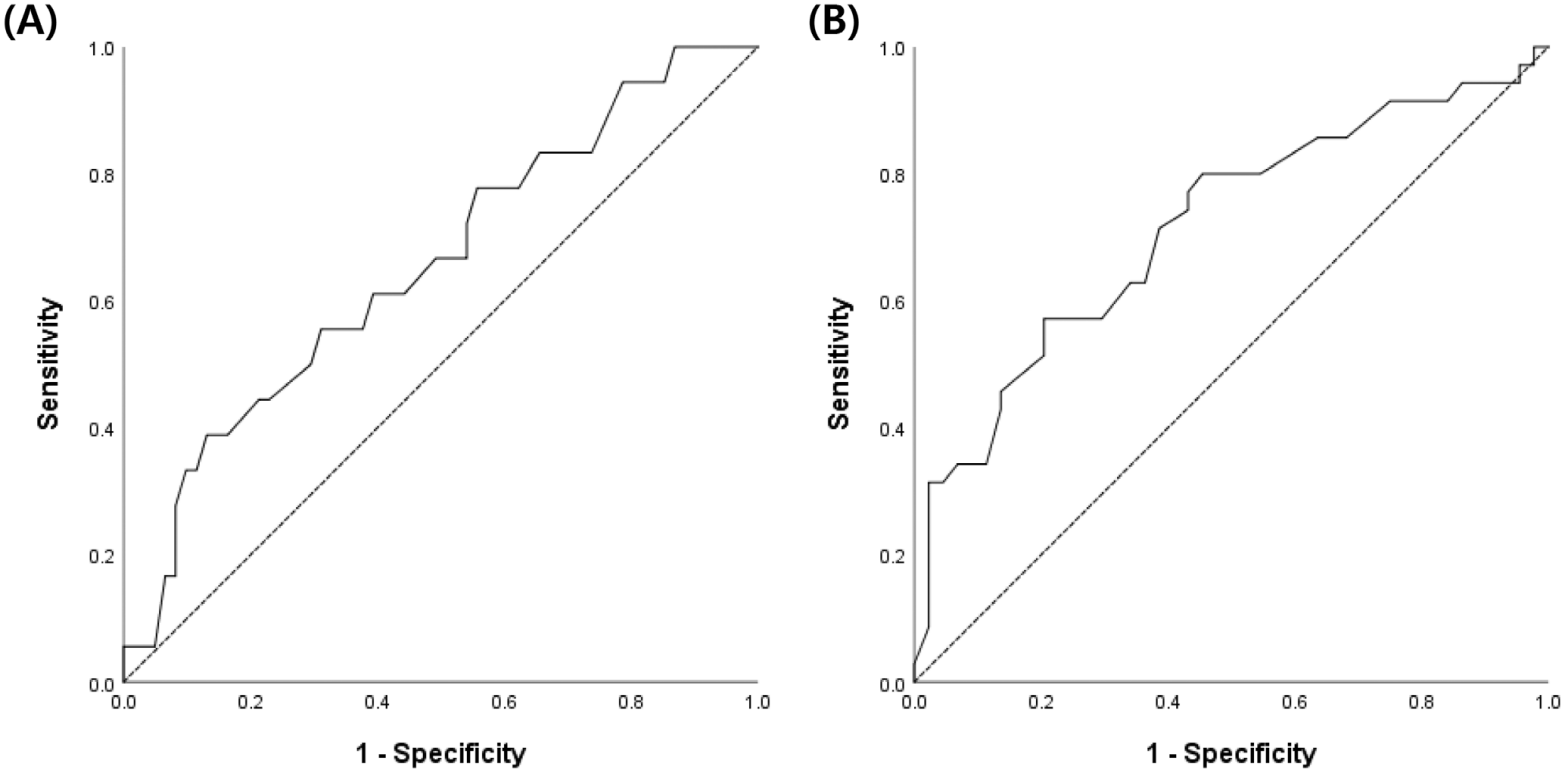
Receiver operating characteristic (ROC) curves for progression‐free survival (PFS) and overall survival (OS) events. (A) ROC curve for PFS events, demonstrating the ability of the delta‐He values to predict PFS. The area under the curve (AUC) is 0.655 with a *p*‐value of 0.047. The optimal cutoff value, determined by the Youden index method, is 3.8 pg, with the second highest cutoff observed at 3.3 pg. (B) ROC curve for OS events, showing the predictive power of the delta‐He values for OS. The AUC is 0.715 with a *p*‐value of 0.001. The Youden index method identifies a cutoff value of 3.3 pg.

In the ROC curve for PFS events, the area under the curve (AUC) was 0.655, with a corresponding *p*‐value of 0.047 (Figure 1A). Employing the Youden index method, the calculated cutoff value was 3.8 pg, with the second highest value observed at 3.3 pg.

Subsequently, ROC analysis for OS yielded an AUC of 0.715 and a *p*‐value of 0.001 (Figure 1B), with a cutoff of 3.3 pg using the Youden index method.

The final cutoff value was established as 3.3 pg by a comprehensive consideration of both analyses. This consolidated approach, considering both PFS and OS, enhanced the robustness and applicability of the determined cutoff value in predicting outcomes in the present study.

### 3.3 Clinical Outcomes According to Delta‐He

Stratifying patients based on delta‐He values (≤ 3.3 pg as Low and > 3.3 pg as High) produced 50 and 29 individuals in the low and high delta‐He groups, respectively (Table 2). Demographic factors, such as age, sex, and smoking history, and disease characteristics, such as stage at diagnosis, cytological features, and PD‐L1 expression, did not differ significantly between the groups.

**TABLE 2:** Clinical characteristics and treatment outcomes according to Delta‐He.

	Low delta‐He (*N* = 50)	High delta‐He (*N* = 29)	*p*‐value
Age	69.9 ± 8.9	70.1 ± 7.7	0.904
Sex			0.492
Male	40 (80.0)	25 (86.2)	
Female	10 (20.0)	4 (13.8)	
Smoking status			0.361
Never	9 (18.0)	3 (10.3)	
Former/Current	41 (82.0)	26 (89.7)	
Disease stage at diagnosis			0.256
IIIA	0 (0.0)	1 (3.4)	
IIIB	4 (8.0)	1 (3.4)	
IIIC	1 (2.0)	2 (6.9)	
IVA	27 (54.0)	19 (65.5)	
IVB	18 (36.0)	6 (20.7)	
IL‐6	27.5 ± 46.0	13.6 ± 13.2	0.053
Cell type			0.671
Squamous carcinoma	19 (38.0)	14 (48.3)	
Adenocarcinoma	29 (58.0)	14 (48.3)	
Other	2 (4.0)	1 (3.4)	
PD‐L1			0.066
High	16 (34.0)	3 (10.3)	
Low	8 (17.0)	6 (20.7)	
No	23 (48.9)	20 (69.0)	
Treatment method			0.001
Monotherapy	43 (86.0)	15 (51.7)	
Combination therapy	7 (14.0)	14 (48.3)	
No. of prior regimens			0.141
0	18 (36.0)	17 (58.6)	
1	28 (56.0)	10 (34.5)	
≥ 2	4 (8.0)	2 (6.9)	
ICI agent			0.491
Atezolizumab	22 (44.0)	9 (31.0)	
Nivolumab	2 (4.0)	2 (6.9)	
Pembrolizumab	26 (52.0)	18 (62.1)	
Number of ICI	8.7 ± 10.3	12.5 ± 8.6	0.095
ICI treatment duration	172.3 ± 221.7	256.7 ± 194.2	0.092
First response			0.021
PR	12 (24.0)	10 (34.5)	
SD	14 (28.0)	15 (51.7)	
PD	23 (46.0)	4 (13.8)	
Best response			0.023
PR	13 (26.0)	12 (41.4)	
SD	13 (26.0)	13 (44.8)	
PD	23 (46.0)	4 (13.8)	
ORR	26.0	41.4	
DCR	52.0	86.2	
irAEs	13 (26.0)	6 (20.7)	0.786
Concomitant RT	14 (28.0)	5 (17.2)	0.413

*Note:* Data are presented as mean ± standard deviation or number (%), unless otherwise indicated.

Abbreviations: DCR, disease control rate; ICI, immune checkpoint inhibitor; IL‐6, interleukin‐6; irAE, immune‐related adverse events; ORR, objective response rate; PD, progressive disease; PD‐L1, programmed death‐ligand 1; PR, partial response; RT, radiation therapy; SD, stable disease.

An analysis of treatment responses revealed notable differences between patients with low delta‐He (≤ 3.3 pg) and high delta‐He (> 3.3 pg) values (Table 2). The assessment of the best response further demonstrated this trend, with the high delta‐He group showing a higher percentage of PR (41.4%) compared to the low delta‐He group (26.0%), resulting in a significant difference (*p*‐value 0.023).

Patients in the low delta‐He group had a significantly poorer prognosis than those in the high delta‐He group. This difference was reflected in objective response rates (ORR) and DCR. The high delta‐He group showed a higher ORR (41.4%) compared to that of the low delta‐He group (26.0%). Moreover, the DCR was significantly higher in the high delta‐He group (86.2%) compared to that in the low delta‐He group (52.0%). Analysis of adverse events (AEs) and treatment characteristics revealed differences between the two groups. The low delta‐He group experienced a slightly higher incidence of AEs (26.0%) compared to the high delta‐He group (20.7%), although this difference did not reach statistical significance.

### 3.4 Statistical Analysis

**FIGURE 2:**
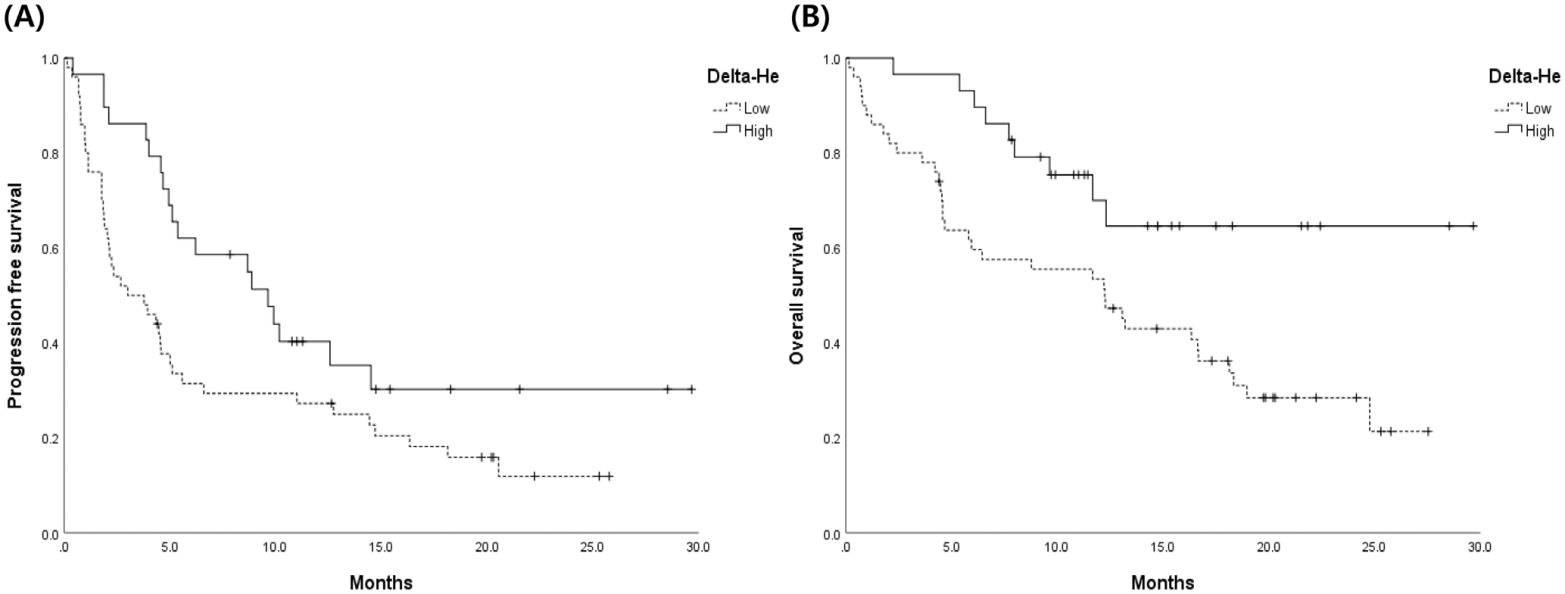
Kaplan–Meier survival curves for progression‐free survival (PFS) and overall survival (OS) in non‐small cell lung cancer (NSCLC) patients based on delta‐He values. (A) Kaplan–Meier curve for PFS. Patients are divided into low and high delta‐He groups. The median PFS for the low delta‐He group is 3.00 months (95% CI: 0.57–5.43), for the high delta‐He group is 9.63 months (95% CI: 5.00–14.27), and for the overall group is 4.93 months (95% CI: 4.08–5.79). The log‐rank test shows a statistically significant difference in PFS distribution between the high and low delta‐He groups (chi‐square = 5.096, *p*‐value 0.024). (B) Kaplan–Meier curve for OS. The median OS for the low delta‐He group is 12.23 months (95% CI: 7.28–17.18), while the median OS for the high delta‐He group is not reached. The delta‐He value for the overall group is 16.33 months (95% CI: 11.43–21.56). The log‐rank test shows a statistically significant difference in OS distribution between the high and low delta‐He groups (chi‐square = 6.671, *p*‐value 0.010).

Kaplan–Meier analysis revealed a distinctive PFS pattern among patients with NSCLC (Figure 2A). Median PFS in the low and high delta‐He groups was 3.00 (95% CI: 0.57–5.43) and 9.63 months (95% CI: 5.00–14.27), respectively, and that of the overall group was 4.93 months (95% CI: 4.08–5.79). The log‐rank test revealed a statistically significant difference in PFS distribution between the high and low delta‐He groups (chi‐square = 5.096, *p*‐value 0.024).

Kaplan–Meier analysis showed distinct OS patterns among patients with lung cancer (Figure 2B). The median OS in the low delta‐He group was 12.23 months (95% CI: 7.28–17.18). The median OS in the high delta‐He group was not reached. The median OS in the overall group was 16.33 months (95% CI: 11.43–21.56). The log‐rank test revealed a statistically significant difference in OS distribution between the high and low delta‐He groups (chi‐square = 6.671, *p*‐value 0.010).

The Cox regression analysis revealed a significant association between the delta‐He values and PFS in patients receiving immunotherapy (Table 3). Particularly, patients with low delta‐He values exhibited a higher risk of mortality (hazard ratio [HR] = 1.853; 95% CI: 1.074–3.199, *p*‐value 0.027) compared to those with high delta‐He levels. Additionally, factors corresponding to lung function such as smoking status (HR = 0.466; 95% CI: 0.237–0.913, *p*‐value 0.026), DM (HR = 1.819; 95% CI: 1.051–3.148, *p*‐value 0.033), and forced expiratory volume in 1 s (FEV1) (HR = 0.984; 95% CI: 0.973–0.995, *p*‐value 0.005) were significantly correlated with PFS.

**TABLE 3:** Cox proportional hazards analysis of factors influencing progression free survival.

	Univariate analysis			Multivariate analysis		
	Hazard ratio	95% CI	*p*‐value	Hazard ratio	95% CI	*p*‐value
Age	0.997	0.966–1.029	0.842			
Delta‐He (low vs. high)	1.853	1.074–3.199	0.027	1.849	01.04—3.284	0.016
Gender (male vs. female)	0.653	0.334–1.275	0.212			
Smoking (ever vs. never)	0.466	0.237–0.913	0.026	0.531	0.265–1.064	0.074
HTN	0.653	0.391–1.091	0.104			
DM	1.819	1.051–3.148	0.033	1.316	0.717–2.418	0.376
Staging	1.152	0.828–1.603	0.401			
FEV1 (%)	0.984	0.973–0.995	0.005	0.985	0.974–0.997	0.015
Adenocarcinoma (vs. squamous)	0.913	0.546–1.526	0.728			
PD‐L1 high (vs. low, no)	2.054	1.052–4.012	0.035	0.089	0.908–3.903	0.089
Driver mutation	0.582	0.263–1.287	0.181			
Combination Tx (vs. MonoTx)	0.623	0.340–1.142	0.126			
IrAE	0.758	0.410–1.402	0.377			
Concomittant RT	0.660	0.357–1.223	0.187			

Abbreviations: DM, diabetes mellitus; FEV1, forced expiratory volume in 1 s; HTN, hypertension; irAE, immune‐related adverse events; MONO, monotherapy; PD‐L1, programmed death‐ligand 1; RT, radiation therapy; Tx, treatment.

The multivariate analysis of the data showed that the low delta‐He group was more likely to have a PFS event than the high delta‐He group (HR = 1.849; *p*‐value 0.036, 95% CI: 1.04–3.284), whereas the HR of PFS in patients with a history of smoking was 0.500 (95% CI: 0.250–0.999, *p*‐value 0.050).

In the Cox regression analysis for OS, the lower delta‐He group exhibited a more than two‐fold increase in the risk of mortality (HR = 2.55; 95% CI: 1.221–5.325, *p*‐value 0.013) compared to the high delta‐He group. Other significant predictors of survival included pulmonary function as measured by FEV1, where lower values were associated with a higher risk of death (HR = 0.428; 95% CI: 0.254–0.722 *p*‐value 0.001). In addition, age (HR = 1.037; 95% CI: 0.997–1.079, *p*‐value 0.068), DM (HR = 1.732; 95% CI: 0.924–3.248, *p*‐value 0.087), and the presence of a driver mutation (HR = 0.376; 95% CI: 0.134–1.055, *p*‐value 0.063) were significant predictors of mortality at a *p*‐value of 0.1 in the univariate analysis.

In the multivariate analysis, low FEV1 (HR 0.984, 95% CI: 0.969–0.999, *p*‐value 0.034) and low delta‐He (HR 2.368, 95% CI: 1.094–5.128, *p*‐value 0.005) were significantly associated with OS (Table 4).

**TABLE 4:** Cox proportional hazards analysis of factors influencing overall survival.

	Univariate analysis			Multivariate analysis		
	Hazard ratio	95% CI	*p*‐value	Hazard ratio	95% CI	*p*‐value
Age	1.037	0.997–1.079	0.068	1.035	0.986–1.086	0.162
Delta‐He (low vs. high)	2.55	1.221–5.325	0.013	2.368	1.094–5.128	0.005
Gender (male vs. female)	0.882	0.408–1.906	0.749			
Smoking (ever vs. never)	0.917	0.407–2.062	0.833			
HTN	1.155	0.639–2.086	0.634			
DM	1.732	0.924–3.248	0.087	1.015	0.494–2.082	0.968
Staging	1.213	0.799–1.841	0.364			
FEV1 (%)	0.428	0.254–0.722	0.001	0.982	0.967–0.997	0.016
Adenocarcinoma (vs. squamous)	0.843	0.460–1.548	0.582			
PD‐L1 High (vs. low, no)	0.502	0.230–1.095	0.083	0.494	0.207–1.179	0.112
Driver mutation	0.376	0.134–1.055	0.063	0.565	0.180–1.775	0.328
Combination Tx (vs. mono)	0.582	0.257–1.319	0.195			
IrAE	0.928	0.468–1.840	0.831			
Concomittant RT	1.076	0.553–2.092	0.829			

Abbreviations: DM, diabetes mellitus; FEV1, forced expiratory volume in 1 s; HTN, hypertension; irAE, immune‐related adverse events; MONO, monotherapy; PD‐L1, programmed death‐ligand 1; RT, radiation therapy; Tx, treatment.

## 4 Discussion

This study suggests that the delta‐He value is a potent predictive and prognostic marker for PFS and OS in patients with NSCLC treated with ICIs. Delta‐He analysis demonstrated high specificity and sensitivity for predicting PFS and OS. High delta‐He values indicated significantly better survival in both PFS and OS.

Based on a comparative analysis of the low and high delta‐He groups, several outcomes showed the potential advantage of the high delta‐He group. Despite similar distributions in baseline characteristics such as age, sex, disease stage, and PD‐L1 expression, outcomes such as ORR and DCR tended to be more favorable in the high delta‐He group. Moreover, in both univariate and multivariate analyses, low delta‐He levels consistently indicated a significantly worse prognosis in both PFS and OS, with a high OR. These results highlight the utility of the delta‐He value as a robust predictive and prognostic indicator in patients with NSCLC undergoing ICI therapy, suggesting its relevance in guiding treatment decisions and predicting patient outcomes.

To date, PD‐L1 expression in cancer cells, as assessed by an immunohistochemical analysis of biopsy specimens, remains the sole biomarker approved by the FDA for predicting responses to ICIs that have been fully implemented in clinical practice [22]. In the present study, PD‐L1 expression did not differ significantly between the high and low delta‐He groups. In the survival analysis, the PFS and OS of the high PD‐L1 expression group showed better survival outcomes than the PD‐L1 low/no expression group, which is consistent with the observations of clinical practice.

The Kaplan–Meier curve distinctly showed that, regardless of the PD‐L1 expression level, the high delta‐He group had superior outcomes compared to the low delta‐He group. Furthermore, the delta‐He value could more clearly predict PFS and OS in the group with low or no PD‐L1 expression than in the group with high PD‐L1 expression (*p*‐value 0.006, 0.010, Figure S1). In the group with high PD‐L1 expression, *p*‐values of 0.169 for PFS and 0.069 for OS were not statistically significant. This is likely due to the fact that there were only two patients with high PD‐L1 expression and high delta‐He values. Both patients had no progression or death events and were still on treatment 15.4 and 21.5 months after ICI initiation. The lack of significant results may be due to the small sample size of the study, and further analyses with a larger sample may yield more significant results.

The patients included in this study are somewhat heterogeneous. Some patients received ICI monotherapy or combination therapy, some had driver mutations, and some received radiotherapy as an add‐on during treatment because ICIs were not effective enough. A total of 58 patients were treated with monotherapy, and 21 patients were treated with combination therapy. Combination therapy may reflect an additive effect of cytotoxic chemotherapy on cancer treatment. In the subgroup analysis, the statistical significance was not strong for both PFS (*p*‐value 0.106 for monotherapy and *p*‐value 0.272 for combination therapy) and OS (*p*‐value 0.077 for monotherapy and *p*‐value 0.094 for combination therapy, Figure S2). Although the small number of patients and the lack of statistical significance make it difficult to draw conclusions based on these data alone, we observed a trend towards a more significant benefit in the high delta‐He group than the low delta‐He group.

Also, there were 13 patients with driver mutations. Driver mutations are known to cause resistance to immunotherapy and predict poor prognosis. Driver mutations are associated with molecular mechanisms that shape the immune tumor microenvironment and may impede ICI therapy [23]. Among the patients with driver mutations, there were 2 cases with EGFR mutations, 7 with KRAS mutations, 1 with ALK rearrangement, 1 with ROS1 mutation, and 1 with BRAF mutation. Due to the limited number of cases for each specific mutation, stratification and subgroup analyses based on individual driver mutations could not be performed. Instead, a subgroup analysis was conducted based on the presence or absence of any driver mutation. In the subgroup analysis, patients with no driver mutation showed statistical significance for OS (*p*‐value 0.004) and no statistical significance but a trend in PFS (*p*‐value 0.064, Figure S3). Future studies with larger patient cohorts will be necessary to allow for more detailed analyses of specific driver mutations and their impact on the predictive and prognostic value of delta‐He. A total of 19 patients received additional radiation therapy as a booster to immunotherapy. Radiation can also affect the treatment effectiveness of ICIs. Patients who had not received radiation therapy also showed better prognosis in high delta‐He and a statistical significance in PFS and OS (*p*‐value 0.044, 0.012, Figure S4). Based on this, we suggest that the value of delta‐He is a good predictive and prognostic marker in patients with NSCLC treated with PD‐1/PD‐L1 inhibitors, excluding other factors that may affect treatment, such as driver mutation or radiotherapy.

The multivariate analysis indicated that baseline pulmonary function test results, specifically, FEV1 and delta‐He values, could be influential in predicting patient outcomes, corroborating previous studies that have shown that an FEV1 value < 80% in pulmonary function tests before the use of ICIs is a predictor of patient outcomes [24]. Although a patient's systemic condition and cancer status are important prior to treatment, the findings suggest that a careful review of coexisting conditions and underlying pulmonary conditions, along with pulmonary function tests such as FEV1, may predict patient's prognosis. This difference was more pronounced in the OS group.

Currently, delta‐He is not a widely used hematologic parameter, but its advantages as a hematologic assay include its noninvasiveness, rapidity, accessibility, and cost‐effectiveness. Although primarily researched in the context of anemia and iron availability and previously applied to predict outcomes in patients undergoing peritoneal dialysis and surgeries, its utility in oncology, particularly lung cancer, presents a novel and significant finding. It is known that IL‐6 induces hepcidin expression, which reduces iron availability and subsequently affects RET‐He levels [25]. Baseline serum IL‐6 levels are potential biomarkers for predicting the efficacy and survival benefits of PD‐1/PD‐L1 inhibitors in NSCLC [26]. The incorporation of delta‐He analysis amidst ongoing investigations into its relationship with hepcidin and inflammation, including IL‐6, suggests its potential as an unmet predictive and prognostic tool for patients with cancer.

The limitations of our study include its relatively small sample size and single‐institution setting, potentially affecting the generalizability of the results. Multicenter studies with larger cohorts and prospective designs are essential to validate the predictive value of delta‐He values. Additionally, the absence of an independent validation cohort and the sole focus on delta‐He, without considering other biomarkers, merits attention in future research. Also, This study did not include a control group of NSCLC patients not receiving immunotherapy, as it was designed to collect samples specifically from patients undergoing immunotherapy. This limits the ability to determine whether delta‐He is exclusively predictive in the context of PD‐1/PD‐L1 inhibitors. Future studies should include a control group of patients receiving alternative treatments, such as chemotherapy or targeted therapy, to clarify the specificity and broader applicability of delta‐He as a predictive and prognostic biomarker.

In summary, baseline delta‐He levels have emerged as a promising predictive and prognostic biomarker for patients with lung cancer undergoing PD‐1/PD‐L1 inhibitor immunotherapy. There is considerable potential to influence lung cancer management by refining therapeutic choices and enhancing patient outcomes. Future research should delve into the mechanistic role of delta‐He values in cancer and its integration into clinical practice, representing advancements in personalized oncology.

## Author Contributions

**Green Hong:** conceptualization (equal), data curation (equal), formal analysis (equal), investigation (equal), methodology (equal), resources (equal), visualization (equal), writing – original draft (equal), writing – review and editing (equal). **Song‐I Lee:** data curation (equal), resources (equal), writing – review and editing (equal). **Da Hyun Kang:** data curation (equal), resources (equal), writing – review and editing (equal). **Chaeuk Chung:** data curation (equal), resources (equal), writing – review and editing (equal). **Hee Sun Park:** conceptualization (equal), data curation (equal), formal analysis (equal), funding acquisition (equal), investigation (equal), methodology (equal), resources (equal), visualization (equal), writing – original draft (equal), writing – review and editing (equal). **Jeong Eun Lee:** conceptualization (equal), data curation (equal), formal analysis (equal), funding acquisition (equal), investigation (equal), methodology (equal), resources (equal), software (equal), visualization (equal), writing – original draft (equal), writing – review and editing (equal).

## Ethics Statement

This study retrospectively reviewed medical records; however, patient samples were collected prospectively in accordance with the Declaration of Helsinki and Good Clinical Practice guidelines, and the study was approved by the Institutional Review Board of CNUH (2020–10‐077).

## Consent

Written informed consent was obtained from all patients prior to their participation in the study.

## Conflicts of Interest

The authors declare no conflicts of interest.

## Supporting information


**Figure S1.** K‐M survival curves for PFS and OS in patients with NSCLC stratified by PD‐L1 expression and delta‐He values. (A) PFS for patients with low/no PD‐L1 expression (chi‐square = 6.695, *p*‐value 0.010). (B) PFS for patients with high PD‐L1 expression (chi‐square = 3.313, *p*‐value 0.069). (C) OS for patients with low/no PD‐L1 expression (chi‐square = 7.656, *p*‐value 0.006). (D) OS for patients with high PD‐L1 expression (chi‐square = 1.890, *p*‐value 0.169).

**Figure S2.** K‐M survival curves for PFS and OS in patients with NSCLC based on delta‐He values and treatment type. (A) PFS for patients receiving monotherapy (chi‐square = 2.610, *p*‐value 0.106). (B) PFS for patients receiving combination therapy (chi‐square = 1.209, *p*‐value 0.272). (C) OS for patients receiving monotherapy (chi‐square = 3.124, *p*‐value 0.077). (D) OS for patients receiving combination therapy (chi‐square = 2.801, *p*‐value 0.094).

**Figure S3.** K‐M survival curves for PFS and OS in patients with NSCLC based on delta‐He values and presence of driver mutation. (A) PFS for patients without driver mutation (chi‐square = 3.431, *p*‐value 0.064). (B) PFS for patients with driver mutation (chi‐square = 1.962, *p*‐value 0.161). (C) OS for patients without driver mutation (chi‐square = 8.212, *p*‐value 0.004). (D) OS for patients with driver mutation (chi‐square = 0.140, *p*‐value 0.708).

**Figure S4.** K‐M survival curves for PFS and OS in patients with NSCLC based on delta‐He values and radiotherapy status. (A) PFS for patients without radiotherapy (chi‐square = 4.071, *p*‐value 0.044). (B) PFS for patients with radiotherapy (chi‐square = 2.002, *p*‐value 0.157). (C) OS for patients without radiotherapy (chi‐square = 6.290, *p*‐value 0.012). (D) OS for patients with radiotherapy (chi‐square = 0.596, *p*‐value 0.440).

## Data Availability

All data generated or analyzed during this study are included in this published article and its in Supporting Information files.
