# Supplementary figures and images for: Delta‐He as a Novel Predictive and Prognostic Biomarker in Patients With NSCLC Treated With PD–1/PD‐L1 Inhibitors
Source: Cancer Med. 2025 Apr 5;14(7):e70826. doi: 10.1002/cam4.70826 (PMC11971532; doi:10.1002/cam4.70826)

Supplementary figure 1

PD-L1 Low/No expression

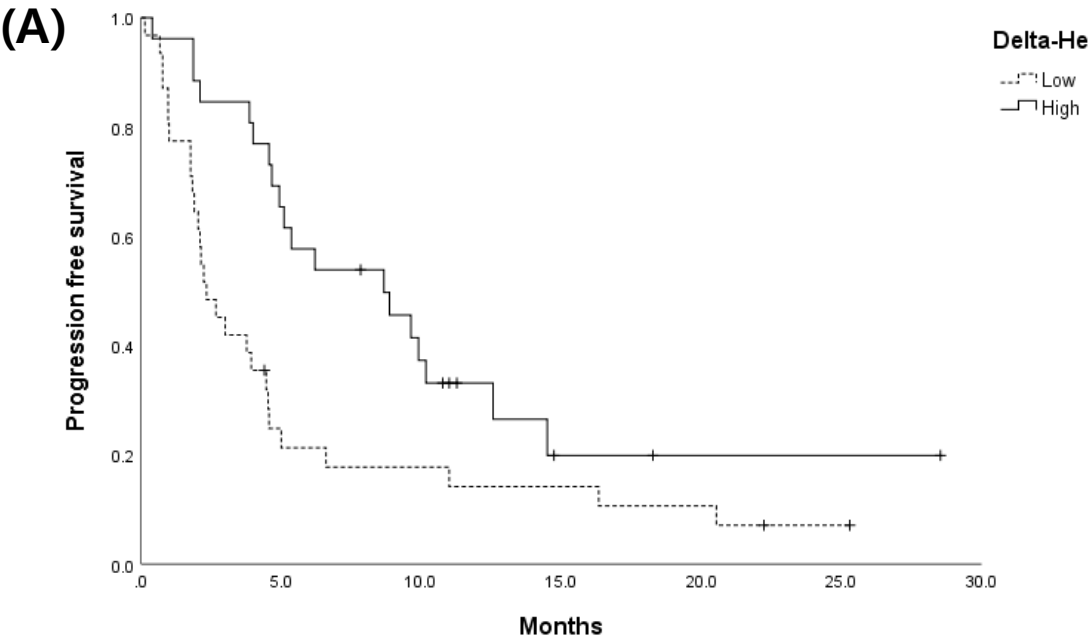

PD-L1 High expression

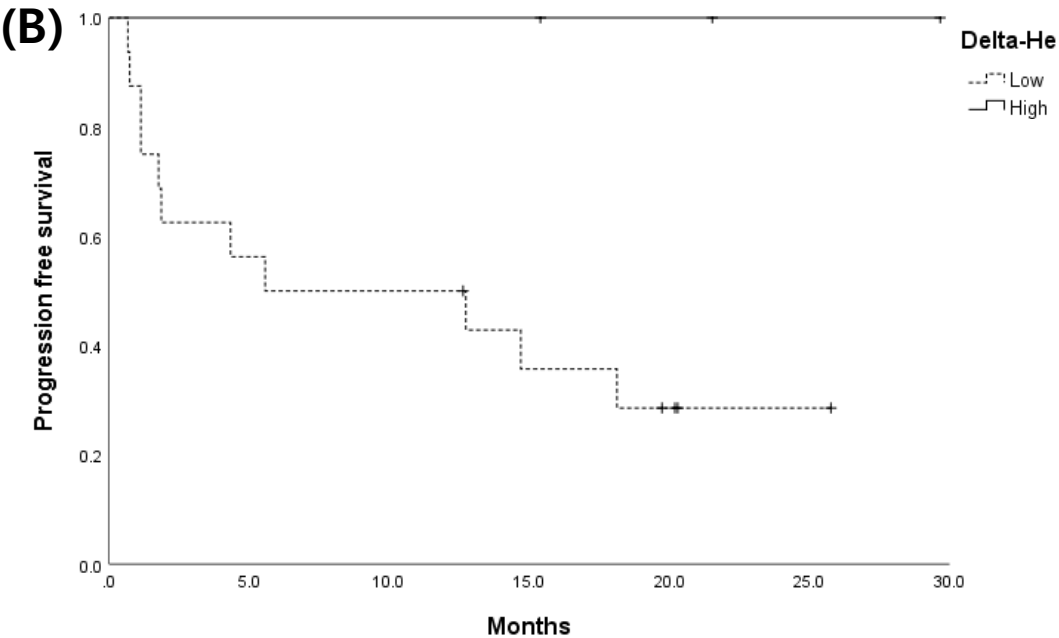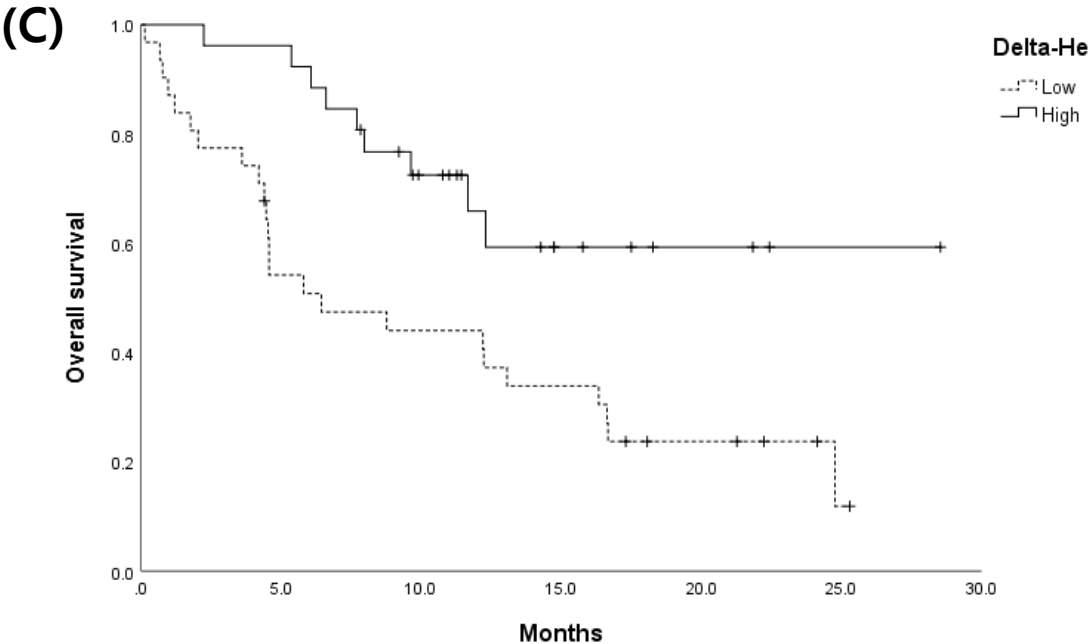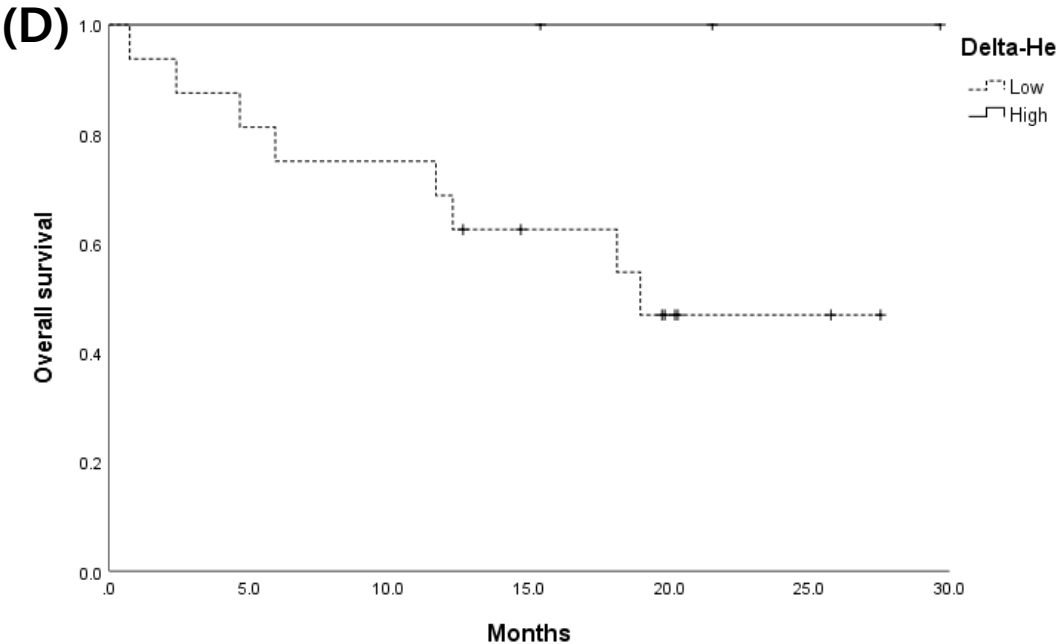

Supplement: Supplementary file 1 — Figure S1. K‐M survival curves for PFS and OS in patients with NSCLC stratified by PD‐L1 expression and delta‐He values. (A) PFS for patients with low/no PD‐L1 expression (chi‐square = 6.695, p‐value 0.010). (B) PFS for patients with high PD‐L1 expression (chi‐square = 3.313, p‐value 0.069). (C) OS for patients with low/no PD‐L1 expression (chi‐square = 7.656, p‐value 0.006). (D) OS for patients with high PD‐L1 expression (chi‐square = 1.890, p‐value 0.169). [file CAM4-14-e70826-s001.pdf]

Supplementary figure 2

(A)

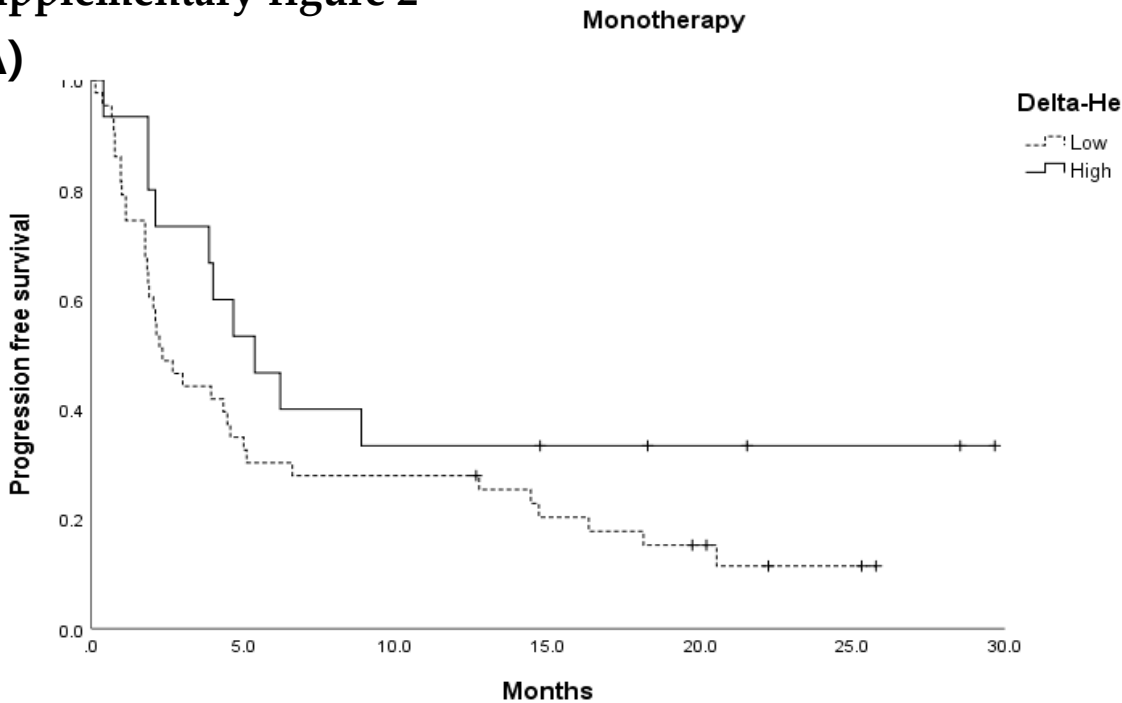

(B)

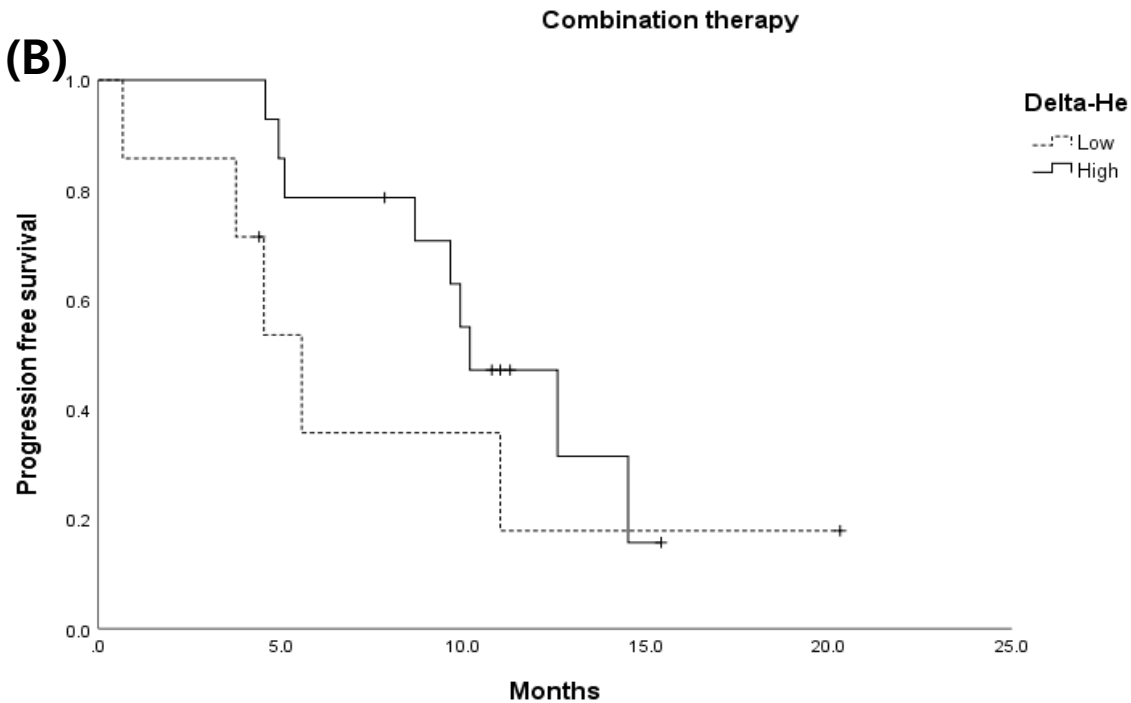

(C)

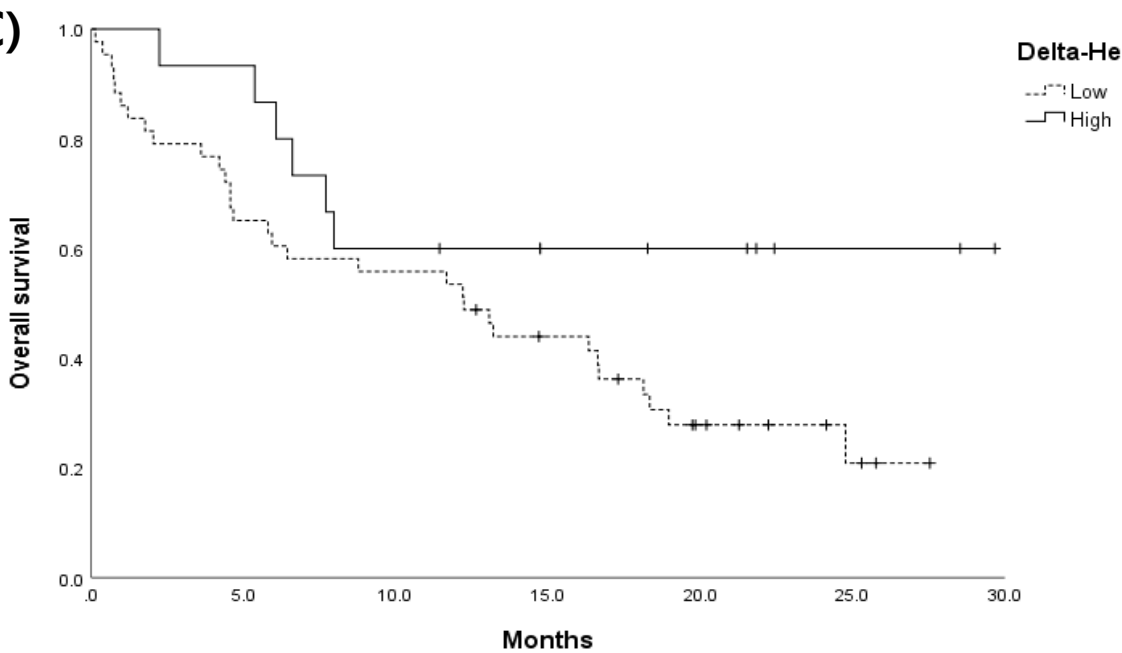

(D)

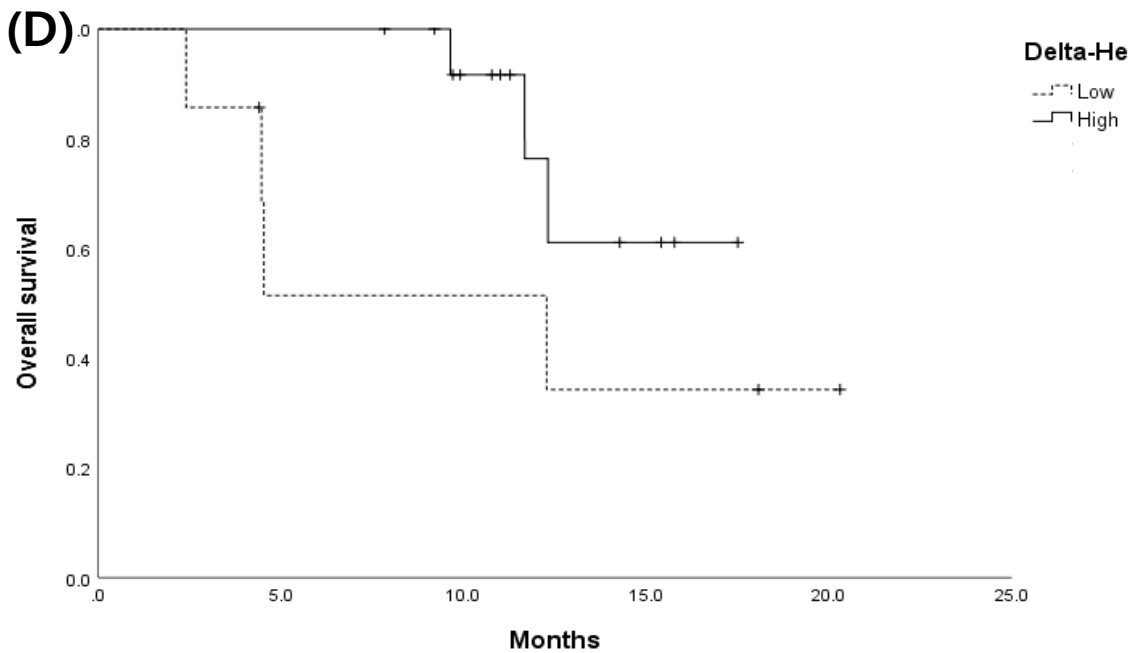

Supplement: Supplementary file 2 — Figure S2. K‐M survival curves for PFS and OS in patients with NSCLC based on delta‐He values and treatment type. (A) PFS for patients receiving monotherapy (chi‐square = 2.610, p‐value 0.106). (B) PFS for patients receiving combination therapy (chi‐square = 1.209, p‐value 0.272). (C) OS for patients receiving monotherapy (chi‐square = 3.124, p‐value 0.077). (D) OS for patients receiving combination therapy (chi‐square = 2.801, p‐value 0.094). [file CAM4-14-e70826-s002.pdf]

Supplementary figure 3

No driver mutation

Driver mutation

(A)

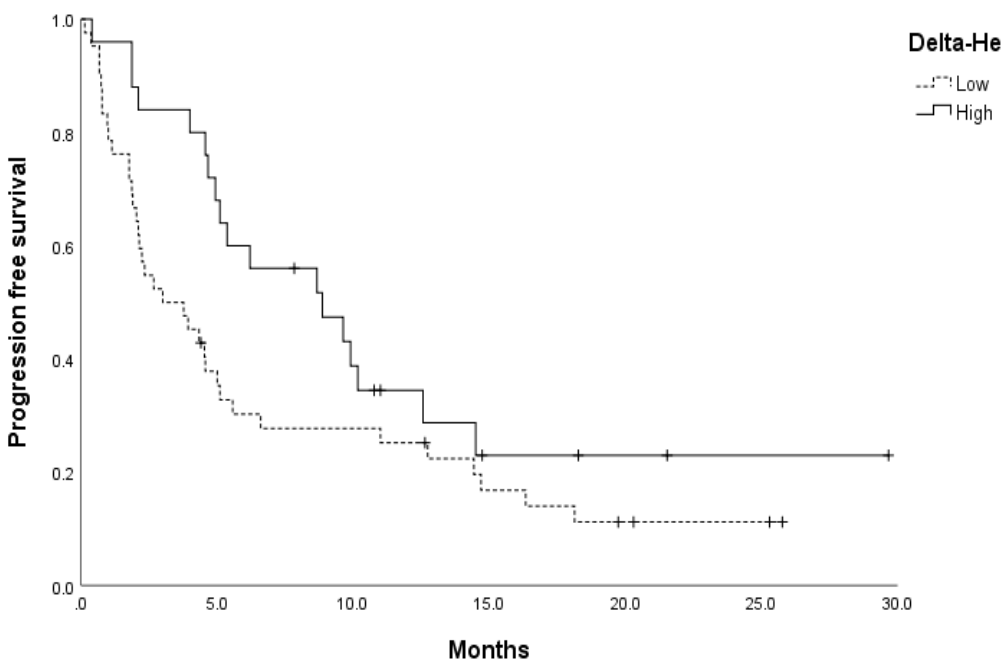

(B)

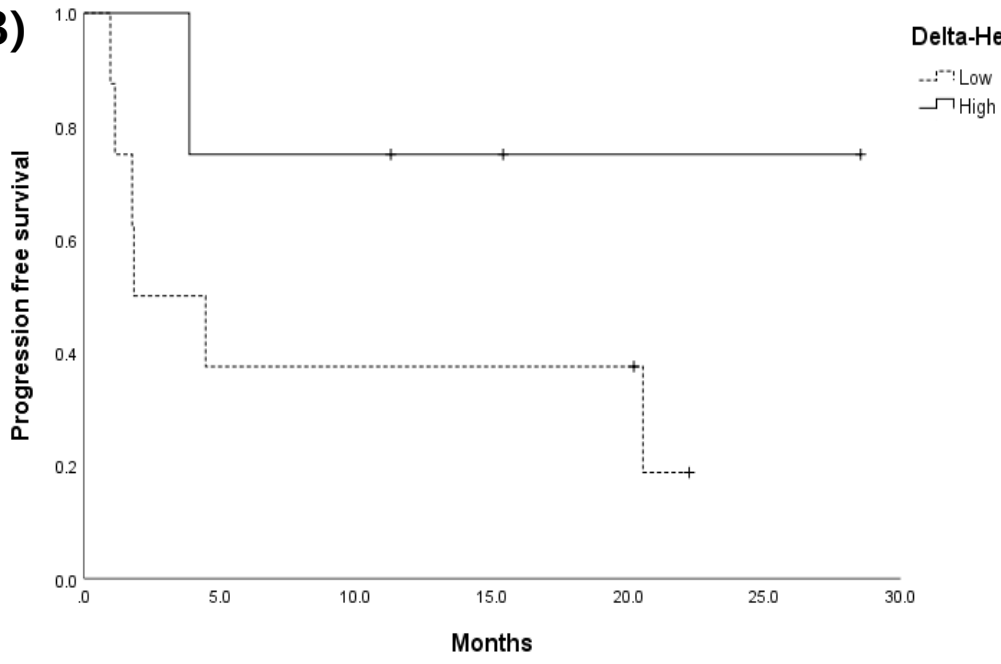

(C)

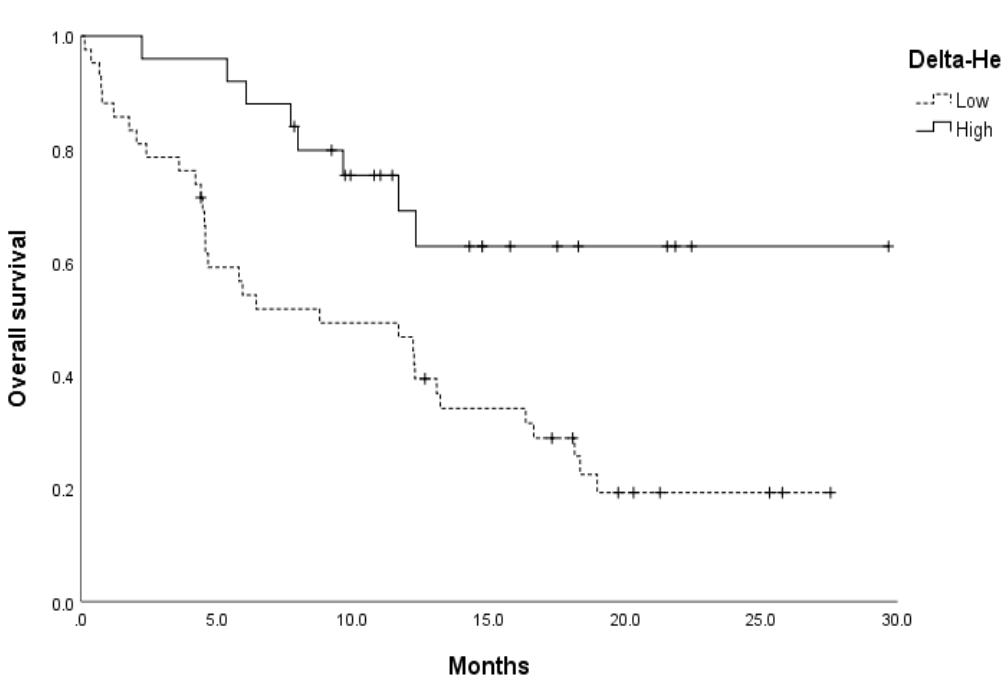

(D)

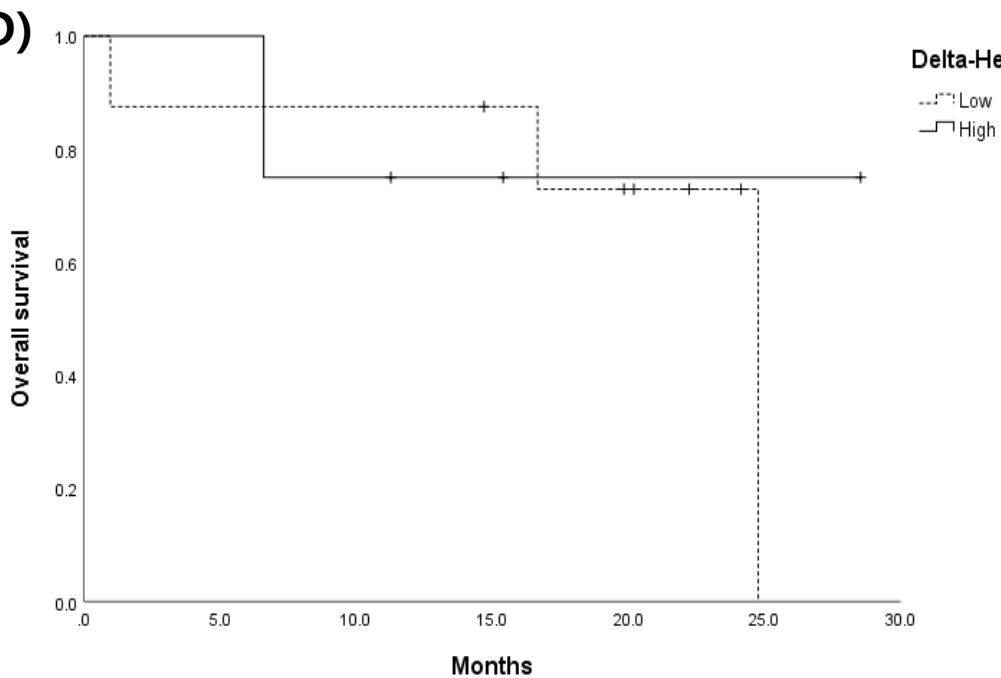

Supplement: Supplementary file 3 — Figure S3. K‐M survival curves for PFS and OS in patients with NSCLC based on delta‐He values and presence of driver mutation. (A) PFS for patients without driver mutation (chi‐square = 3.431, p‐value 0.064). (B) PFS for patients with driver mutation (chi‐square = 1.962, p‐value 0.161). (C) OS for patients without driver mutation (chi‐square = 8.212, p‐value 0.004). (D) OS for patients with driver mutation (chi‐square = 0.140, p‐value 0.708). [file CAM4-14-e70826-s004.pdf]

Supplementary figure 4

No radiotherapy

Radiotherapy

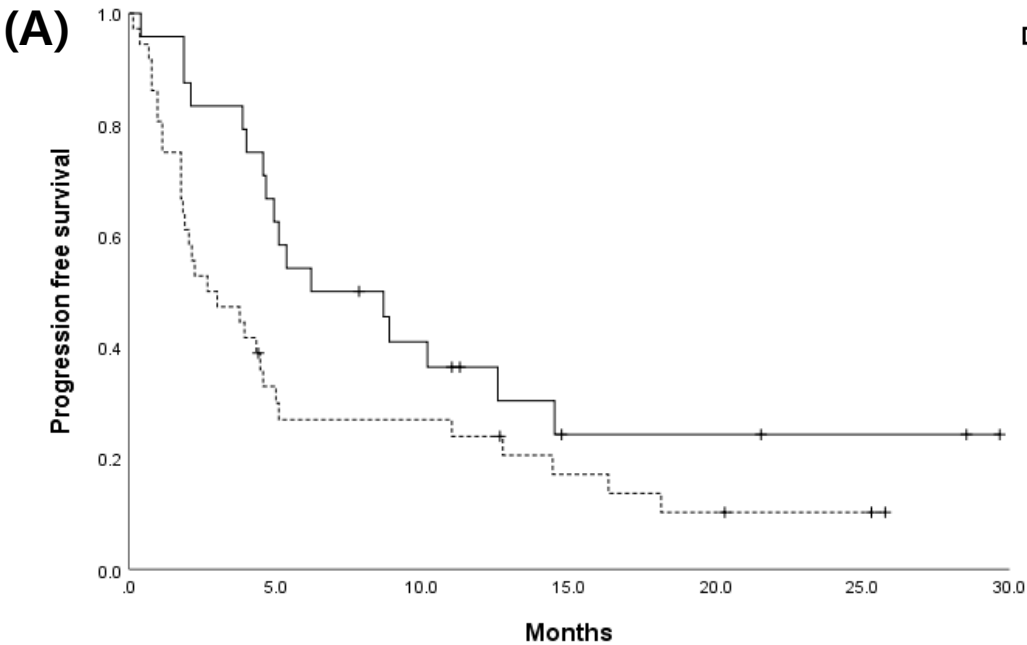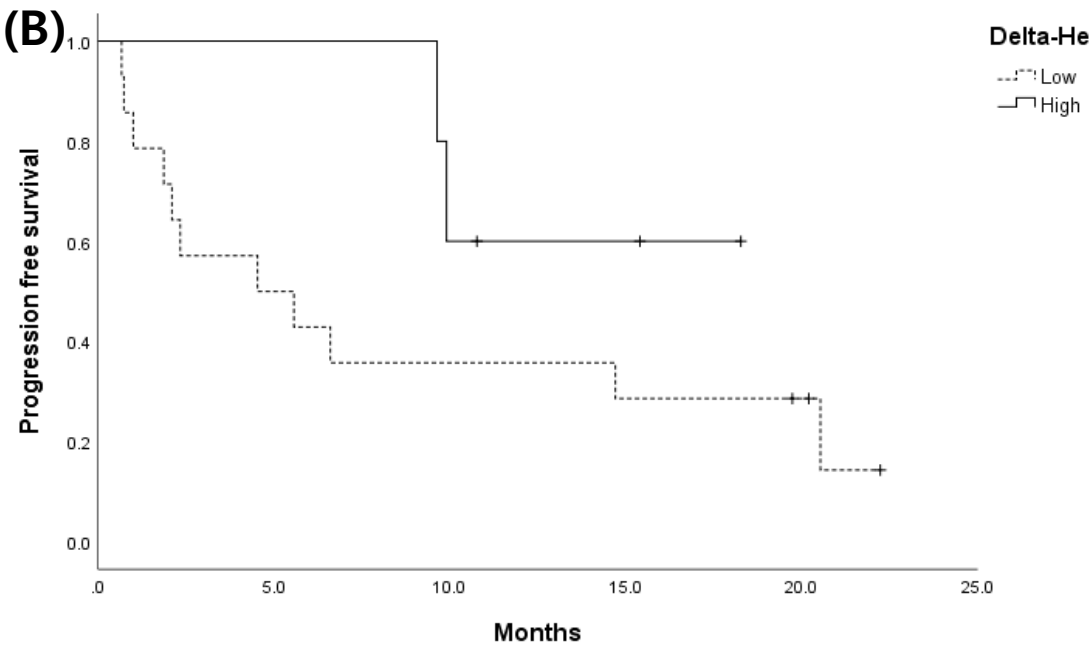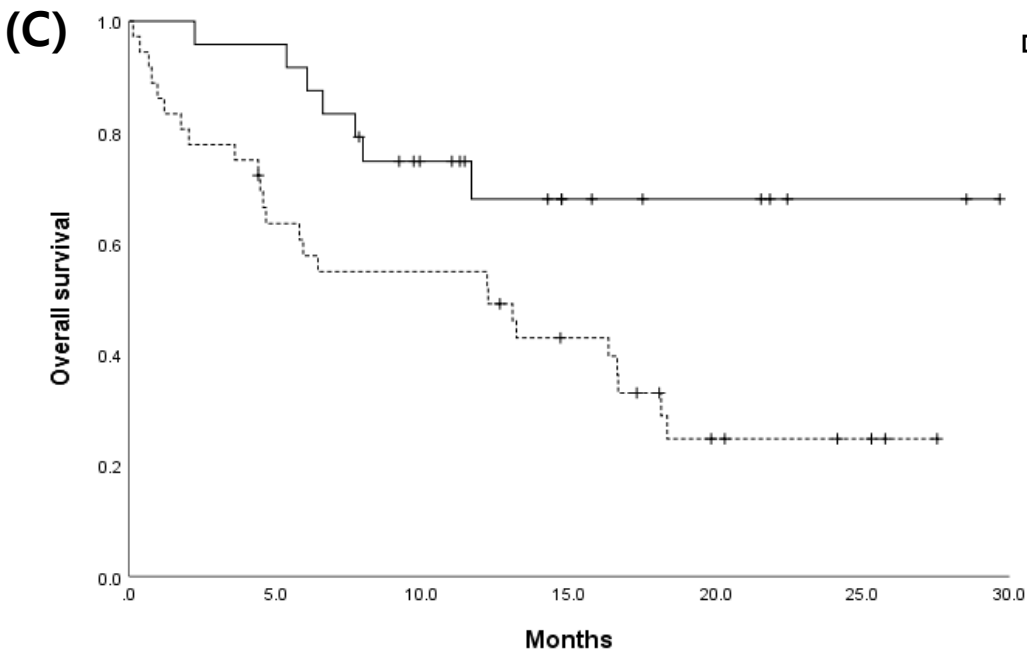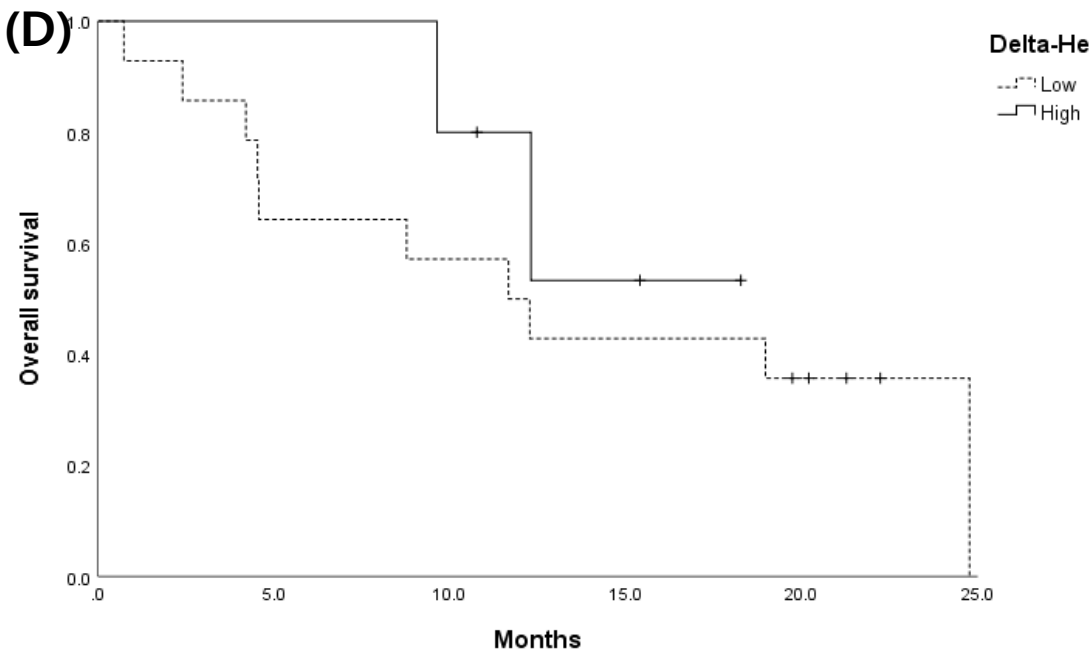

Supplement: Supplementary file 4 — Figure S4. K‐M survival curves for PFS and OS in patients with NSCLC based on delta‐He values and radiotherapy status. (A) PFS for patients without radiotherapy (chi‐square = 4.071, p‐value 0.044). (B) PFS for patients with radiotherapy (chi‐square = 2.002, p‐value 0.157). (C) OS for patients without radiotherapy (chi‐square = 6.290, p‐value 0.012). (D) OS for patients with radiotherapy (chi‐square = 0.596, p‐value 0.440). [file CAM4-14-e70826-s003.pdf]
